# A case with a trend of QT interval prolongation due to the introduction of methadone to a pancreatic cancer patient on levofloxacin

**DOI:** 10.1186/s40780-023-00322-w

**Published:** 2024-01-02

**Authors:** Ryusuke Ouchi, Munenori Nagao, Shinju Suzuki, Toshihiro Yamagata, Mie Chiba, Naoko Kurata, Kensuke Usui, Takashi Watanabe, Kaori Koyama, Kouji Okada

**Affiliations:** 1 Division of Clinical Pharmaceutics and Pharmacy Practice, Faculty of Pharmaceutical Sciences, Tohoku Medical and Pharmaceutical University, Sendai, Miyagi Japan; 2 Department of Pharmacy, Tohoku Medical and Pharmaceutical University Hospital, Sendai, Miyagi Japan; 3 Department of Supportive Medicine and Care for Cancer, Tohoku Medical and Pharmaceutical University Hospital, Sendai, Miyagi Japan; 4 Department of Nursing, Tohoku Medical and Pharmaceutical University Hospital, Sendai, Miyagi Japan

**Keywords:** Cancer pain, Drug interactions, Levofloxacin, Methadone, QT interval prolongation

## Abstract

**Background:**

As methadone can prevent the development of opioid resistance, it has application in alleviating cancer-related pain that proves challenging to manage with other opioids. QT interval prolongation is a serious side effect of methadone treatment, with some reported deaths. In particular, owing to the increased risk of QT interval prolongation, caution should be exercised when using it in combination with drugs that also prolong the QT interval.

**Case presentation:**

This study presents a case in which methadone was introduced to a patient (a man in his 60s) already using levofloxacin, which could prolong the QT interval—a serious side effect of methadone treatment—and whose QTc value tended to increase. Given that levofloxacin can increase the risk of QT interval prolongation, we considered switching to other antibacterial agents before introducing methadone. However, because the neurosurgeon judged that controlling a brain abscess was a priority, low-dose methadone was introduced with continuing levofloxacin. Owing to the risks, we performed frequent electrocardiograms. Consequently, we responded before the QTc increased enough to meet the diagnostic criteria for QT interval prolongation. Consequently, we prevented the occurrence of drug-induced long QT syndrome.

**Conclusions:**

When considering the use of methadone for intractable cancer pain, it is important to eliminate possible risk factors for QT interval prolongation. However, as it may be difficult to discontinue concomitant drugs owing to comorbidities, there could be cases in which the risk of QT interval prolongation could increase, even with the introduction of low-dose methadone. In such cases, frequent monitoring, even with simple measurements such as those used in this case, is likely to prevent progression to more serious conditions.

## Background

Methadone is an opioid analgesic that acts on μ receptors and blocks N-methyl-D-aspartate receptors [1]. As methadone can prevent the development of opioid resistance, it is used for alleviating cancer pain that is difficult to treat with other opioids. However, because a clear conversion ratio with other opioids has not been established, the blood concentration half-life is very long and there are large individual differences [2], it is sometimes difficult to determine the methadone dosage at introduction. In addition, QT interval prolongation is a serious side effect of methadone treatment [3, 4], and there have been some reported deaths [5]. In particular, owing to the increased risk of QT interval prolongation [6], caution is required when using it in combination with drugs that also prolong the QT interval.

We present a case in which methadone was introduced to a patient already using levofloxacin (LVFX), which is known to have a risk of prolonging the QT interval [7], and whose QTc value tended to increase. In recent years, the advances in anticancer treatment have resulted in longer treatment periods and an increase in comorbidities and concomitant drugs owing to the aging of cancer survivors [8]. Therefore, when patients use methadone for cancer pain, it may prove problematic to use it in combination with drugs that can prolong the QT interval. We believe that this case is useful as a countermeasure when the concomitant use of drugs with a risk of QT interval prolongation cannot be avoided at the introduction of methadone.

## Case presentation

The patient was a man in his 60s who visited the Department of Gastroenterology for the treatment of autoimmune pancreatitis and had been diagnosed with pancreatic cancer with liver metastasis (T2N0M1, stage IV) two years previously. Subsequently, he consulted the Department of Medical Oncology and started chemotherapy, which included four courses of FOLFIRINOX, 10 courses of GEM + nab-PTX, and two courses of nal-IRI + 5-FU/LV. As his case was judged to be a progressive disease one year prior, the hospital focused on palliative care. At that time, he was referred to the Department of Supportive Medicine and Care for Cancer because of severe abdominal pain, which was considered cancer-related, and administration of 10 mg/day of oxycodone was started. Thereafter, he continued outpatient visits, and the oxycodone dose was adjusted according to the pain level. However, three months prior, he was admitted to the Department of Neurosurgery because he developed a brain abscess associated with infection after surgery for a cerebral aneurysm. After subcutaneous irrigation and drainage, his symptoms improved, and he was discharged from hospital. He continued to receive LVFX 500 mg/day to prevent recurrence of brain abscess. Subsequently, the dose of oxycodone was increased to 50 mg/day for worsening abdominal pain by the outpatient clinic, but it was still difficult to control the pain; therefore, he was admitted to the Department of Supportive Medicine and Care for Cancer for pain control. At the time of admission, his height was 169.0 cm and body weight was 63.5 kg; he was ambulatory and capable of all self-care but unable to perform any work activities (performance status 2; denoting the day of admission as day 1).

**Fig. 1:**
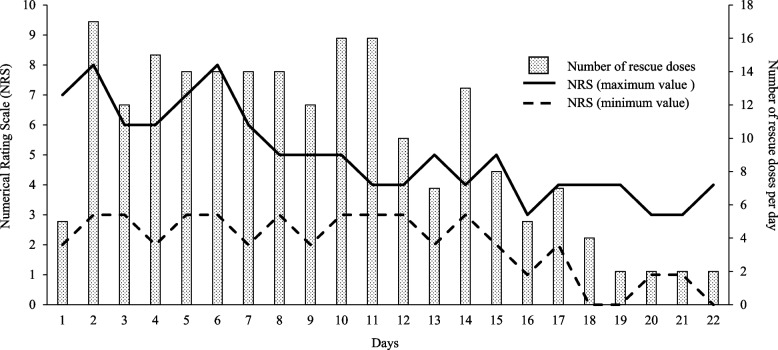
Changes in the number of rescue doses and numerical rating scale. The number of rescue doses is shown from 0:00 to 24:00. For the scale, the maximum and minimum values for the day are shown

**Fig. 2:**
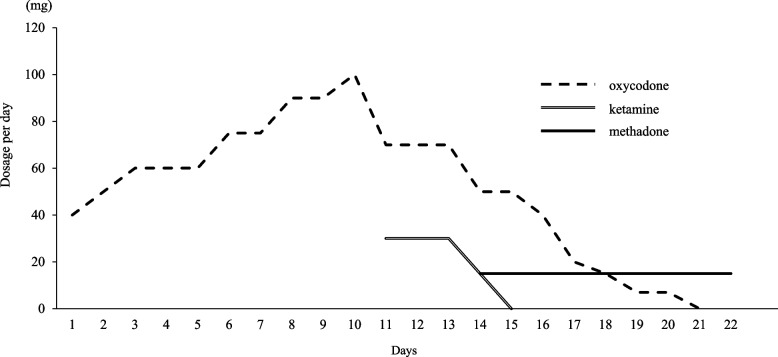
Dose transition of narcotic analgesics. Dosage trends for oxycodone, ketamine, and methadone are shown. Oxycodone and ketamine were injectable preparations and methadone was administered orally

**Fig. 3:**
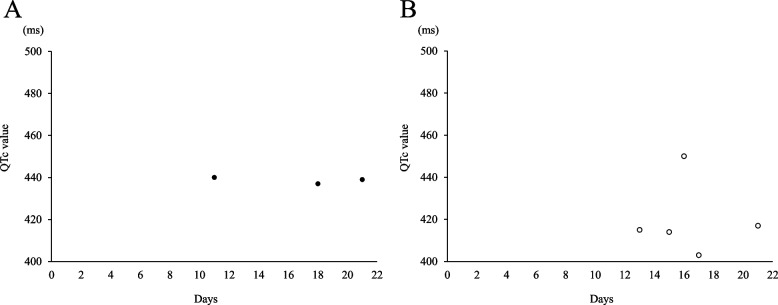
Changes in QTc value. For QTc, the results of the 12-lead (**A**) and simple (**B**) electrocardiogram are shown. Because the two forms of electrocardiogram were measured using different devices, changes in QTc were judged according to different criteria

**Fig. 4:**
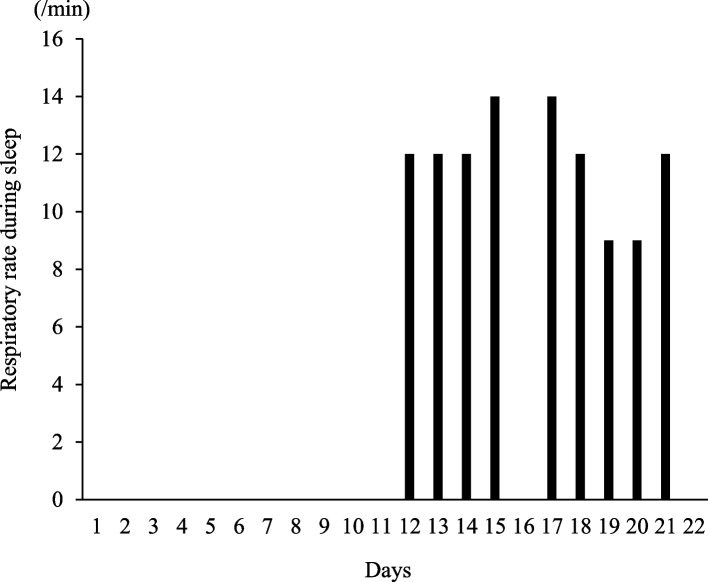
Changes in respiratory rate. Regarding the change in respiratory rate, the number of breaths counted during sleep is shown. The respiratory rate on day 16 is not shown because it was not recorded

Upon admission, hepatic dysfunction and exacerbation of inflammation were observed. In addition, CT examination indicated exacerbation of the primary tumor, enlargement of the liver metastases, and appearance of multiple lung metastases; therefore, the primary disease was judged to have aggravated rapidly. At the time of admission, he complained of severe pain with a numerical rating scale (NRS) score of 7/10 (Fig. 1). Therefore, he was switched to an injection of oxycodone 40 mg/day, and the dose had to be adjusted to 100 mg/day by day 10. However, the pain persisted, with an NRS score of 5/10, and ketamine 30 mg/day was initiated because of the poor response to increasing opioid doses. Several hours after the start of the ketamine treatment, the patient complained of nausea. Therefore, the oxycodone dose was reduced to 70 mg/day and the nausea improved. The day after the start of the ketamine treatment, an improvement to NRS = 3–4/10 and a decrease in the number of rescue doses was observed; methadone was introduced because the patient and his family wanted him to be discharged. Methadone treatment (15 mg/day) was initiated on day 14. The doses of oxycodone and ketamine were tapered and terminated (Fig. 2). In addition, we considered that LVFX, which carries a risk of QT interval prolongation, has to be discontinued or changed; however, the neurosurgeon recommended the continuation of LVFX because it showed no evidence of the recurrence of brain abscess. Additionally, bacterial culture showed that the causative bacteria of the brain abscess were very sensitive to LVFX. Therefore, the patient’s QTc was carefully monitored, along with his respiratory rate (Figs. 3 and 4).

Oxycodone was reduced to 50 mg/day and ketamine was reduced to 15 mg/day when methadone was started on day 14. Ketamine was discontinued on day 15 because he did not feel worsening pain. The oxycodone dose was subsequently reduced to 40 mg/day on day 16. On the same day, although it did not meet the diagnostic criteria for long QT syndrome, an increase in QTc of approximately 10% compared to baseline was observed. Therefore, we asked the neurosurgeon to change LVFX to sulfamethoxazole/trimethoprim (ST). On day 17, QTc improved, and the pain subsided (NRS = 2–4/10); therefore, the oxycodone dose was reduced to 20 mg/day. On day 18, oxycodone dose was further reduced to 15 mg/day; on day 19, it was again reduced to 7 mg/day and ended on day 21. Further, no recurrence of the brain abscess was observed by continuing with ST. As he did not experience pain exacerbation (NRS = 1–3/10) on day 22, he was discharged with a dose of methadone of 15 mg/day. Figure 1 shows the changes in the NRS and the number of rescue doses during hospitalization. In addition, Table 1 shows the changes in laboratory values after methadone treatment was initiated, and Table 2 shows a list of the drugs used during hospitalization.

**Table 1:** Results of blood sampling tests

Inspection item	Inspection unit	Day 14^a^	Day 16^a^	Day 22^a^
total bilirubin	mg/dL	2.15	2.64	4.14
aspartate aminotransferase	U/L	139	112	176
alanine aminotransferase	U/L	81	94	120
lactate dehydrogenase	U/L	212	189	280
alkaline phosphatase	U/L	912	926	1147
γ-glutamyl transpeptidase	U/L	2306	2394	2119
blood urea nitrogen	mg/dL	18	16	16
serum creatinine	mg/dL	0.6	0.6	0.82
uric acid	mg/dL	4.2	4.2	4.9
estimated glomerular filtration rate	mL/min/1.73m^2^	100.6	100.6	71.5
albumin	g/dL	2.3	2.4	2.4
Na	mEq/L	132	131	130
K	mEq/L	4.4	4.3	4.8
Cl	mEq/L	94	94	95
Ca	mg/dL	8.8	8.9	8.8
Mg	mg/dL	1.9	1.9	2.0

^a^The day of starting methadone (Day 14), the 3rd day (Day 16), and the 8th day (Day 22) are shown

**Table 2:** List of drugs used during hospitalization

Oral Medicine	Dosage and Administration Schedule
Methadone Hydrochloride Tablets 5 mg	3 Tablets, three times a day, 8:00 a.m., 2:00 p.m., and 9:00 p.m.
Levofloxacin Hydrate Tablets 500 mg	1 Tablet, once a day, after dinner
Dapagliflozin Propylene Glycolate Hydrate Tablets 10 mg	1 Tablet, once a day, after breakfast
Mirogabalin Besilate Tablets 10 mg	2 Tablets, twice a day, after breakfast and dinner
Mirogabalin Besilate Tablets 5 mg	2 Tablets, twice a day, after breakfast and dinner
Vonoprazan Fumarate Tablets 20 mg	1 Tablet, once a day, after breakfast
* Clostridium Butyricum* Tablets	3 Tablets, three times a day, after each meal
Naldemedine Tosilate Tablets 0.2 mg	1 Tablet, once a day, after breakfast
Linaclotide Tablets 0.25 mg	1 Tablet, once a day, before breakfast
Silodosin Tablets 4 mg	2 Tablets, twice a day, after breakfast and dinner
Acetaminophen Tablets 500 mg	4 Tablets, four times a day, after each meal and at bedtime
Lactulose Jelly 12 g	4 Packets, twice a day, after breakfast and dinner
Sulfamethoxazol / Trimethoprim Combination Tablets	4 Tablets, twice a day, after breakfast and dinner
Injection	Dosage per day
Electrolyte solution for infusion (maintenance solution)	500 mL / day (Administration was completed on Day 15)
Ketamine Hydrochloride Injection	30 mg / day (Administration was completed on Day 14)
Amino acids, sugar, electrolytes, vitamin B1 liquid	500 mL / day (Administration was completed on Day 16)
Oxycodone Hydrochloride Hydrate Injection	The dose was gradually reduced and administration was completed on Day 21
Dexamethasone Sodium Phosphate Injection	3.3 mg / day (Administration was completed on Day 16)

## Discussion and conclusions

Owing to its high clinical efficacy, methadone is used for intractable cancer pain for which other opioid analgesics are not sufficiently effective; however, it is likely that QT interval prolongation is a serious side effect [3, 4, 9]. High-dose methadone administration and electrolyte imbalances have been listed as risk factors for QT interval prolongation [10], and testing these is thus recommended when starting methadone [11]. In addition, the concomitant use of drugs with a risk of QT interval prolongation increases the risk of QT interval prolongation due to methadone [6]. In fact, Winton et al. reported a case of QT interval prolongation due to a combination of methadone and azithromycin [12].

The PubMed database (MeSH terms: methadone and levofloxacin) did not include reports on QT interval prolongation in combination with methadone for LVFX, which was administered to prevent brain abscess recurrence. Because LVFX can increase the risk of QT interval prolongation [7], we considered switching to other antibacterial agents before introducing methadone. However, we continued with LVFX. Although this was not a high-dose methadone administration case, because it was used in combination with drugs that have a risk of QT interval prolongation, we assumed that the risk of QT interval prolongation could have increased and performed frequent electrocardiograms (ECGs). A 12-lead ECG was performed once every seven days for routine examination. In addition, a pharmacist took simple ECG measurements using a health monitor (Checkme ProX, SAN-EI MEDISYS, Japan). Based on the results of the simple ECG, we performed an additional 12-lead ECG test in addition to the regular examination if there was a possibility of QT interval prolongation when the QTc value was 500 ms or more, or when the QTc was increased by 25% or more from baseline (QTc before methadone introduction) [9]. Before the introduction of methadone, QTc was within the normal range on both the 12-lead ECG and simple ECG. However, on day 3, after initiating methadone in combination with LVFX (day 16), a simple ECG revealed an 8.4% QTc increase from baseline (Fig. 3). Although the criteria for performing an emergency 12-lead ECG were not met, QTc tended to be prolonged in combination with a drug with a risk of QT interval prolongation. At this time, no decrease in respiratory rate during sleep was observed; therefore, we judged that the prolonged QTc was not derived from methadone overdosage (Fig. 4). Therefore, we considered it necessary to switch from the LVFX to another antibiotic. Considering the results of the bacterial culture of pus during brain abscess treatment, we changed LVFX to ST for preventing the recurrence of the brain abscess. Subsequently, a simple ECG on day 17 improved QTc to the same level as the baseline, and a 12-lead ECG on day 18 did not show an increase in QTc (Fig. 3). As such, we responded before the QTc increased enough to meet the diagnostic criteria for QT interval prolongation and prevent the occurrence of drug-induced long QT syndrome and torsades de pointes.

Other risk factors for QT interval prolongation in this case may include abnormal laboratory values and other concomitant medications. Hypokalemia, hypocalcemia, and hypomagnesemia are factors known to increase the risk of QT interval prolongation due to methadone; however, these abnormalities were not observed in tests conducted before and after starting methadone (Table 1). The risk of QT interval prolongation with concomitant medications other than methadone and LVFX has been reported in ST [13, 14], Lactulose [15], and Vonoprazan [16] (Table 2). Regarding QT interval prolongation due to ST, mutations in MinK-related peptide 1, a subunit of the cardiac potassium channel involved in hereditary long QT syndrome, are involved, and the effect is negligible in the wild type [14]. In addition, QTc decreased after changing from LVFX to ST, which suggests that our judgment was valid. Yuan et al. reported an increase in QTc due to lactulose, but they noted that this may be due to the effects of severe liver dysfunction in the patient’s background, rather than the effect of lactulose itself [15]. Therefore, it is difficult to think that the QT interval prolongation in this case was caused by lactulose. There exists a case report regarding the effect of Vonoprazan on QT interval prolongation [16]. However, in a phase I randomized trial of Vonoprazan, it did not affect the QT/QTc interval in healthy participants, its safety for the heart was confirmed [17], and it is unlikely to be considered a typical side effect. Therefore, we posit that the effect on QTc increase was negligible in this case. No marked decrease in renal function, which is the main excretion route of LVFX and is an indicator of dosage, was observed. Therefore, the QTc increase was considered unlikely to be due to LVFX overdose (Table 1). Regarding liver function, which is thought to affect the metabolism of methadone, an increase above the standard value was observed before starting methadone due to the influence of a metastatic liver tumor (Table 1). However, as mentioned above, no significant decrease in respiratory rate was observed during sleep onset; therefore, although it is possible that methadone metabolism had some effect, the increase in QTc is unlikely to be due to methadone overdose. Further, when the interaction between methadone and LVFX was evaluated using the Drug Interaction Probability Scale (DIPS) [18], the DIPS score was “4” and judged as “Possible.” Considering this in conjunction with the clinical course, the QTc increase in this case may be owing to the combined use of methadone and LVFX, which improved by switching to ST.

When considering the use of methadone for intractable cancer pain, it is important to eliminate possible risk factors for QT interval prolongation as much as possible. However, as it may be difficult to discontinue concomitant drugs owing to comorbidities, there may be cases in which the risk of QT interval prolongation could increase, even with the introduction of low-dose methadone. In such cases, frequent monitoring, even with simple measurements, such as those used in this case, is likely to detect adverse events at an early stage and prevent progression to more serious conditions.

## Data Availability

Data sharing is not applicable to this article as no datasets were generated or analyzed during the current study.

## Abbreviations

LVFX: Levofloxacin
NRS: Numerical rating scale
ST: Sulfamethoxazole/trimethoprim
ECGs: Electrocardiograms
DIPS: Drug Interaction Probability Scale
